# Public perspectives on body and organ donation in Türkiye: barriers, motivations, and strategies for awareness

**DOI:** 10.3389/fpubh.2026.1801282

**Published:** 2026-04-13

**Authors:** Ayla Tekin, Nurşen Zeybek, Yüsra Nur Şanlıtürk, Merve Mergen Şener, Aslıhan Akpınar, Tuncay Çolak

**Affiliations:** 1 Department of Anatomy, Faculty of Medicine, Kocaeli University, Kocaeli, Türkiye; 2 Department of Physiotherapy and Rehabilitation, Faculty of Health Sciences, Istanbul Okan University, Istanbul, Türkiye; 3 Department of Physiotherapy and Rehabilitation, Faculty of Health Sciences, Yalova University, Yalova, Türkiye; 4 Department of History of Medicine and Ethics, Faculty of Medicine, Kocaeli University, Kocaeli, Türkiye

**Keywords:** body donation, cadaver, medical education, organ donation, public perception

## Abstract

**Introduction:**

Body and organ donation are crucial for transplantation and medical education, yet donation rates in Türkiye remain low. This study aimed to explore public perceptions of donation, focusing on demographic factors, motivations, and barriers.

**Methods:**

A cross-sectional survey was conducted from November 2023 to May 2024 using snowball sampling. A total of 16,738 individuals (born 1945–2009) completed an online questionnaire that included demographic variables and 17 items on attitudes toward donation. Data were analyzed using descriptive statistics and Pearson’s chi-square tests with effect size calculations.

**Results:**

The sample was predominantly women (63.1%) and from Generation Z (70%). Overall, 58.8% of participants expressed willingness to donate organs, whereas 18.2% considered body donation. The most common motivator for organ donation was saving lives (74.7%), and the primary reason for body donation was contributing to medical education (67%). The main barriers were concern over bodily integrity for organ donation (41%) and discomfort with exposing the body for body donation (50.1%). Statistically significant associations were found between willingness to donate and variables such as gender, education, and generation (all *p <* 0.001), though effect sizes were small. Despite widespread recognition of the importance of donation, actual willingness, especially for body donation, remains limited.

**Discussion:**

The results showed the need for targeted educational initiatives, clear communication of religious rulings, and institutional transparency to address misconceptions. Given the sampling method and participant profile, the results should be interpreted with caution and not generalized to the entire population.

## Introduction

Body and organ donations play distinct yet interconnected roles in healthcare and education. While organ donations address urgent medical needs through transplantation, body donations aid in the training and development of future healthcare professionals. However, the global demand for both organs and cadavers continues to exceed supply, posing significant challenges for healthcare systems and medical education (1, 2).

According to the World Health Organization, organ shortages remain a universal issue, with thousands of patients on transplant waiting lists worldwide. Similarly, the procurement of cadavers for medical schools is crucial for anatomical studies, yet the availability often falls short of the increasing demand, particularly in countries like Türkiye (3). In Türkiye, the rapid growth in medical faculties, now numbering 128 institutions, has increased the need for cadavers, leaving many institutions reliant on models for anatomy education. This reliance restricts the hands-on experience essential for medical training.

While virtual dissection technologies offer innovative learning tools, they cannot replicate the tactile experience and anatomical variability provided by cadaver-based training, which remains indispensable for developing clinical skills (4, 5). Therefore, the need to promote body donation is becoming increasingly critical.

Despite rising awareness campaigns, donation rates in Türkiye remain low, underscoring the need to examine public perceptions, barriers, and motivations. Misconceptions about organ and body donation, coupled with cultural and religious sensitivities, hinder participation. While organ donation campaigns often succeed through education and media, similar efforts for body donation are underdeveloped.

This study aims to understand public attitudes toward organ and body donation in Türkiye. By identifying barriers, motivations, and strategies, it seeks to offer practical recommendations to increase donation rates through culturally sensitive and trust-building approaches.

## Methods

### Study design

This study used a cross-sectional, descriptive, and exploratory design to investigate public perspectives on body and organ donation in Türkiye. Data were collected between October 2023 and March 2024 using an online questionnaire created via Google Forms.

### Ethical approval and informed consent

This study was approved by the Kocaeli University Non-Interventional Clinical Research Ethics Committee (Approval date: 05/10/2023, decision no: KÜ GOKAEK-2023/16.20, Project no: 2023/326).

All participants provided electronic informed consent before completing the survey. After reading the study purpose and confidentiality statement on the first page of the questionnaire, participants voluntarily confirmed their willingness to participate by clicking the consent checkbox and proceeding with the survey.

The study did not specifically target individuals under 18 years of age. During data collection, it was observed that a small number of 16–17-year-old participants voluntarily and anonymously completed the survey. This situation was reported to the Ethics Committee, which approved retaining these responses for analysis because participation was not actively solicited, no identifying information was collected, and informed consent had been electronically obtained. The youngest participant was born in 2009, meaning all respondents were at least 16 years old at the time of data collection. No participants under the age of 16 were included; therefore, parental or guardian consent was not required.

### Participant recruitment

The survey link was initially shared with a core group comprising university students, academic staff, healthcare professionals, and community members from different regions of Türkiye. A snowball sampling strategy was used to reach hard-to-reach groups and promote widespread participation in nationwide online surveys. To minimize selection bias, the survey was disseminated multiple times across diverse social networks, including both academic and non-academic communities. Participants born between 1945 and 2009 were eligible to participate.

### Survey content

The questionnaire was developed based on a review of previous studies on organ and body donation in Türkiye and internationally (6–9), and the items were adapted and reformulated to fit the aims of the present study. The instrument consisted of 25 items, including eight demographic questions and 17 items addressing motivations, barriers, and recommendations related to donation. The questionnaire included a combination of Likert-type, dichotomous (yes/no), and multiple-choice items.

The instrument was not designed as a unidimensional psychometric scale but instead as a descriptive tool to explore multiple dimensions of public perspectives. However, internal consistency analysis was conducted for the five Likert-type attitudinal items included in the questionnaire. The Cronbach’s alpha coefficient was 0.769, indicating acceptable internal consistency. Item–total correlations were all above 0.30, and no increase in Cronbach’s alpha was observed upon deletion of any item, showing that all items contributed positively to the internal consistency.

Participants were not directly asked to specify their religious affiliations. However, in the section assessing reasons for refusal, the option “I would not donate due to religious reasons” was included. Given that the overwhelming majority of the Turkish population identifies as Muslim, this item was interpreted within the framework of Islamic beliefs.

The questionnaire was developed by adapting and reformulating items from previously published studies and did not use any copyrighted or proprietary instrument; therefore, no formal permission was required.

### Pilot testing and validation

Before data collection, the survey was piloted with 50 individuals to evaluate clarity, comprehensibility, and linguistic consistency. Minor wording adjustments were made accordingly. Content validity was established through expert review by anatomists and medical education specialists, and face validity was supported through pilot testing. Furthermore, the items were derived from previously published studies, further supporting their conceptual relevance.

### Data collection and analysis

The online survey ensured standardized administration, with all participants responding to the same questions under identical conditions. Responses were collected anonymously to reduce social desirability bias, and all items were mandatory to minimize missing data and improve data completeness. This standardized approach contributed to the methodological rigor and reliability of the data collection process.

Responses were exported to Microsoft Excel (Microsoft Corporation, Redmond, WA, USA) and analyzed using IBM SPSS Statistics for Windows, Version 25.0 (IBM Corp., Armonk, NY, USA). Descriptive statistics, including frequencies and percentages, were calculated to summarize demographic characteristics and response distributions. Associations between categorical variables were initially assessed with Pearson’s Chi-square test (*χ*^2^). Statistical significance was set at *p <* 0.05. Effect sizes (ES) were calculated using the formula *ϕ* = √(*χ*^2^/N), where N represents the total sample size. Effect sizes were interpreted according to Cohen’s criteria: 0.10–0.30 (small), 0.30–0.50 (medium), and >0.50 (large). To identify independent predictors of organ donation pledging, body donation pledging, and willingness outcomes, multivariable logistic regression analyses were performed. Variables considered theoretically relevant or significant in univariate analyses were entered into the models using the enter method. Adjusted odds ratios (OR) with 95% confidence intervals (CI) were calculated. Model fit was assessed using the Hosmer–Lemeshow goodness-of-fit test, and overall model significance was evaluated using the Omnibus test of model coefficients. The explanatory power of the models was estimated using Cox & Snell and Nagelkerke R^2^ values.

## Results

### Sample characteristics

A total of 16,738 participants were included in this study. The sample comprised predominantly women participants (*n =* 10,557, 63.1%), reflecting potential gender differences in attitudes toward donation. Most participants were from Generation Z (*n =* 11,715, 70.0%), followed by Generation Y (*n =* 3,371, 20.1%). In terms of education, most participants had completed high school (*n =* 9,197, 54.9%), whereas a significant proportion had graduated from university (*n =* 4,996, 29.8%). Notably, participants with higher education levels exhibited a greater willingness to donate, as shown in the statistical analyses. Most respondents were not health professionals (*n =* 14,659, 87.6%), although a significant number reported having a health professional relative (*n =* 9,836, 58.8%).

### Statistical analysis of body donation willingness

The chi-square analyses showed that several demographic variables were significantly associated with willingness to donate one’s body after death, although the effect sizes were generally small.

Gender showed a statistically significant relationship (*χ*^2^ = 12.128, *p <* 0.001), with men slightly more willing to donate than women. However, the effect size (ES = 0.026) indicated a very weak association (Table 1). Generational differences were more pronounced (*χ*^2^ = 53.801, *p <* 0.001). Younger participants, particularly those in Generation Z, reported a higher willingness to donate compared with older cohorts. The effect size (ES = 0.056) was still small but stronger than that of gender, suggesting generational attitudes may influence donation decisions. Educational attainment also had a significant influence (*χ*^2^ = 35.443, *p <* 0.001). Willingness to donate tended to increase with higher levels of education, ranging from only 12.5% among primary school graduates to nearly 29% among doctoral graduates. Although the effect size was modest (ES = 0.046), the consistent upward trend highlights the importance of education in shaping attitudes toward body donation. Marital status emerged as one of the strongest predictors (*χ*^2^ = 60.198, *p <* 0.001). Single participants expressed a greater willingness to donate than married individuals, with a small-to-moderate effect size (ES = 0.059). Similarly, parenthood was associated with reduced willingness to donate (*χ*^2^ = 47.364, *p <* 0.001), as those without children were more inclined to consider body donation. Conversely, chronic illness (*χ*^2^ = 2.337, *p* = 0.126) and being a health professional (*χ*^2^ = 1.461, *p* = 0.227) were not significantly associated with willingness to donate. This finding suggests that personal health status and professional medical training may not strongly influence body donation decisions. Finally, having a health professional relative showed a weak but significant effect (*χ*^2^ = 6.670, *p* = 0.010; ES = 0.019), with participants who had relatives in healthcare being slightly more willing to donate.

**Table 1:** Demographic variables of participants and willingness to body donation.

Characteristics	*N* (%)	Would you consider body donation after death?		*χ* ^2^	*P* (ES)
		Yes	No		
Gender					
Men	6,181 (36.9%)	1,211 (19.9%)	4,970 (80.4%)	12.128	<0.001 (0.026)
Women	10,557 (63.1%)	1841 (17.4%)	8,716 (82.6%)		
Generations					
Baby boomers (1946–1964)	121 (0.7%)	23 (19.0%)	98 (81.0%)	53.801	<0.001 (0.056)
Generation X (1965–1980)	1,531 (9.1%)	218 (14.2%)	1,313 (85.8%)		
Generation Y (1981–1996)	3,371 (20.1%)	510 (15.1%)	2,861 (84.9%)		
Generation Z (1997–2009)	11,715 (70%)	2,301 (19.6%)	9,414 (80.4%)		
Last graduation					
Primary School	662 (4%)	83 (12.5%)	579 (87.5%)	35.443	<0.001 (0.046)
Secondary School	1,321 (7.9%)	257 (19.5%)	1,064 (80.5%)		
High School	9,197 (54.9%)	1754 (19.1%)	7,443 (80.9%)		
University	4,996 (29.8%)	837 (16.8%)	4,159 (83.2%)		
Master	464 (2.8%)	93 (20.0%)	371 (80.0%)		
PhD	98 (0.6%)	28 (28.6%)	70 (71.4%)		
Marital status					
Married	4,396 (26.3%)	631 (14.4%)	3,765 (85.6%)	60.198	<0.001 (0.059)
Single	12,342 (73.7%)	2,421 (19.6%)	9,921 (80.4%)		
Have children					
Yes	3,854 (23%)	558 (14.5%)	3,296 (85.5%)	47.364	<0.001 (0.053)
No	12,884 (77%)	2,494 (19.4%)	10,390 (80.6%)		
Have a chronic disease					
Yes	1955 (11.7%)	381 (19.5%)	1,574 (80.5%)	2.337	0.126 (0.011)
No	14,783 (88.3%)	2,671 (18.1%)	12,112 (81.9%)		
Are you a health professional					
Yes	2079 (12.4%)	399 (19.2%)	1,680 (80.8%)	1.461	0.227 (0.009)
No	14,659 (87.6%)	2,653 (18.1%)	12,006 (81.9%)		
Have a health professional relative					
Yes	9,836 (58.8%)	1857 (18.9%)	7,979 (81.1%)	6.670	0.01 (0.019)
No	6,902 (41.2%)	1,195 (17.3%)	5,707 (82.7%)		
Total	16,738 (100%)	3,052 (18.0%)	13,686 (82.0%)		

### Statistical analysis of organ donation willingness

A significant association was found between gender and willingness to donate organs (*χ*^2^ = 201.822, *p <* 0.001, ES = 0.109). Women participants (63.0%) reported higher willingness than men (51.8%) (Table 2).

**Table 2:** Demographic variables of participants and willingness to donate organs.

Characteristics	*N* (%)	Would you consider organ donation after death?		*χ* ^2^	*P* (ES)
		Yes	No		
Gender					
Men	6,181 (36.9%)	3,199 (51.8%)	2,982 (48.2%)	201.822	<0.001 (0.109)
Women	10,557 (63.1%)	6,646 (63.0%)	3,911 (37.0%)		
Generations					
Baby boomers (1946–1964)	121 (0.7%)	54 (44.6%)	67 (55.4%)	89.180	<0.001 (0.072)
Generation X (1965–1980)	1,531 (9.1%)	760 (49.6%)	771 (50.4%)		
Generation Y (1981–1996)	3,371 (20.1%)	1908 (56.6%)	1,463 (43.4%)		
Generation Z (1997–2009)	11,715 (70%)	7,123 (60.8%)	4,592 (39.2%)		
Last graduation					
Primary School	662 (4%)	245 (37.0%)	417 (63.0%)	233.555	<0.001 (0.118)
Secondary School	1,321 (7.9%)	639 (48.4%)	682 (51.6%)		
High School	9,197 (54.9%)	5,520 (60.0%)	3,677 (40.0%)		
University	4,996 (29.8%)	3,055 (61.1%)	1941 (38.9%)		
Master	464 (2.8%)	309 (66.6%)	155 (33.4%)		
PhD	98 (0.6%)	77 (78.6%)	21 (21.4%)		
Marital status					
Married	4,396 (26.3%)	2,315 (52.7%)	2081 (47.3%)	93.296	<0.001 (0.074)
Single	12,342 (73.7%)	7,530 (61.0%)	4,812 (39.0%)		
Have children					
Yes	3,854 (23%)	1973 (51.2%)	1881 (48.8%)	120.169	<0.001 (0.084)
No	12,884 (77%)	7,872 (61.1%)	5,012 (38.9%)		
Have a chronic disease					
Yes	1955 (11.7%)	1,121 (57.3%)	834 (42.7%)	1.997	0.158 (0.010)
No	14,783 (88.3%)	8,724 (59.0%)	6,059 (41.0%)		
Are you a health professional					
Yes	2079 (12.4%)	1,371 (66.0%)	708 (34.0%)	49.778	<0.001 (0.054)
No	14,659 (87.6%)	8,474 (57.8%)	6,185 (42.2%)		
Have a health professional relative					
Yes	9,836 (58.8%)	5,995 (60.9%)	3,841 (39.1%)	44.733	<0.001 (0.051)
No	6,902 (41.2%)	3,850 (55.8%)	3,052 (44.2%)		
Total	16,738 (100%)	9,845 (58.8%)	6,893 (41.2%)		

Generational differences were also significant (*χ*^2^ = 89.180, *p <* 0.001, ES = 0.072). Willingness to donate was reported by 44.6% of Baby Boomers, 49.6% of Generation X, 56.6% of Generation Y, and 60.8% of Generation Z. Education level showed a strong relationship with donation willingness (*χ*^2^ = 233.555, *p <* 0.001, ES = 0.118). The percentages increased progressively from primary school graduates (37%) to secondary school (48.4%), high school (60%), university (61.1%), master’s (66.6%), and PhD (78.6%). Marital status was significantly associated with willingness (*χ*^2^ = 93.296, *p <* 0.001, ES = 0.074). Willingness was reported by 52.7% of married participants and 61% of single participants. Having children was significantly associated with willingness (*χ*^2^ = 120.169, *p <* 0.001, ES = 0.084). Those without children (61.1%) were more willing to participate compared to those with children (51.2%). Chronic disease status did not show a significant association (*χ*^2^ = 1.997, *p* = 0.158, ES = 0.010). Being a health professional was significantly associated with willingness (*χ*^2^ = 49.778, *p <* 0.001, ES = 0.054). Among health professionals, 66% expressed willingness compared to 57.8% of non-professionals. Having a health-professional relative was also significantly related (*χ*^2^ = 44.733, *p <* 0.001, ES = 0.051). Willingness was reported by 60.9% of participants with a health-professional relative, compared with 55.8% of those without such a relative.

### Univariate analysis of factors associated with body donation pledging

Of the participants, 391 individuals (2.3%) reported pledging to donate their bodies, whereas 16,347 (97.7%) stated that they had not. Gender differences were statistically significant (*χ*^2^ = 36.034, *p <* 0.001, ES = 0.046), with men (3.3%) reporting body donation more frequently than women (1.8%). Generational analysis also revealed significant differences (*χ*^2^ = 8.589, *p* = 0.036, ES = 0.022), with the highest proportion of body donation observed among Generation Y, whereas baby boomers reported the lowest rate (Table 3).

**Table 3:** Sample characteristics of those who have donated bodies.

Characteristics	*N* (%)	Have you pledged to donate your body?		*χ* ^2^	*P* (ES)
		Yes	No		
Gender					
Men	6,181 (36.9%)	201 (3.3%)	5,980 (96.7%)	36.034	<0.001 (0.046)
Women	10,557 (63.1%)	190 (1.8%)	10,367 (98.2%)		
Generations					
Baby boomers (1946–1964)	121 (0.7%)	3 (2.5%)	118 (97.5%)	8.589	0.036 (0.022)
Generation X (1965–1980)	1,531 (9.1%)	37 (2.4%)	1,494 (97.6%)		
Generation Y (1981–1996)	3,371 (20.1%)	101 (3%)	3,270 (97%)		
Generation Z (1997–2009)	11,715 (70%)	250 (2.1%)	11,465 (97.9%)		
Last graduation					
Primary School	662 (4%)	33 (5.0%)	629 (95.0%)	34.436	<0.001 (0.045)
Secondary School	1,321 (7.9%)	27 (2.0%)	1,294 (98.0%)		
High School	9,197 (54.9%)	206 (2.2%)	8,991 (97.8%)		
University	4,996 (29.8%)	103 (2.1%)	4,893 (97.9%)		
Master	464 (2.8%)	15 (3.2%)	449 (96.8%)		
PhD	98 (0.6%)	7 (7.1%)	91 (92.9%)		
Marital status					
Married	4,396 (26.3%)	133 (3.0%)	4,263 (97.0%)	12.422	<0.001 (0.027)
Single	12,342 (73.7%)	258 (2.1%)	12,084 (97.9%)		
Have children					
Yes	3,854 (23%)	110 (2.9%)	3,744 (97.1%)	5.893	0.015 (0.018)
No	12,884 (77%)	281 (2.2%)	12,603 (97.8%)		
Have a chronic disease					
Yes	1955 (11.7%)	67 (3.4%)	1888 (96.6%)	11.551	0.001 (0.026)
No	14,783 (88.3%)	324 (2.2%)	14,459 (97.8%)		
Are you a health professional					
Yes	2079 (12.4%)	77 (3.7%)	2002 (96.3%)	19.464	0.001 (0.034)
No	14,659 (87.6%)	314 (2.1%)	14,345 (97.9%)		
Have a health professional relative					
Yes	9,836 (58.8%)	239 (2.4%)	9,597 (97.6%)	0.921	0.337 (0.007)
No	6,902 (41.2%)	152 (2.2%)	6,750 (97.8%)		
Total	16,738 (100%)	391 (2.3%)	16,347 (97.7%)		

Educational background was strongly associated with body donation (*χ*^2^ = 34.436, *p <* 0.001, ES = 0.045). While only a small proportion of participants had donated across all education groups, the relative frequency was higher among PhD graduates (7.1%) compared to those with lower levels of education. Marital status also showed a significant association (*χ*^2^ = 12.422, *p <* 0.001, ES = 0.027), with single participants reporting donation more often than married onesSimilarly, participants without children donated at a higher rate than those with children (*χ*^2^ = 5.893, *p* = 0.015, ES = 0.018). A significant association was also found between the presence of chronic disease and body donation (*χ*^2^ = 11.551, *p* = 0.001, ES = 0.026), with higher donation rates reported among those with chronic illness. Being a health professional was another factor significantly related to body donation (*χ*^2^ = 19.464, p = 0.001, ES = 0.034), as health professionals were more likely to have donated than non-health professionals. Conversely, having a health-professional relative did not show a statistically significant relationship with body donation (*χ*^2^ = 0.921, *p* = 0.337, ES = 0.007).

### Multivariable analysis of factors associated with body donation pledging

Multivariable logistic regression analysis was performed to identify independent predictors of body donation pledging (Table 4). The overall model was statistically significant (*χ*^2^ = 103.051, *p <* 0.001). After adjustment, men were associated with lower odds of body donation pledging (OR = 0.506, *p <* 0.001). Higher educational level showed a partial association, with university graduates having lower odds compared to PhD holders (OR = 0.426, *p* = 0.044). Married participants were less likely to pledge body donation than single individuals (OR = 0.667, *p* = 0.041). Similarly, having a chronic disease was associated with reduced odds of pledging (OR = 0.662, *p* = 0.004). Conversely, being a health professional was associated with significantly higher odds of body donation pledging (OR = 1.863, *p <* 0.001). Generation, having children, and having a health professional relative were not significant predictors in the multivariable model (*p* > 0.05).

**Table 4:** Multivariable logistic regression analysis of factors associated with body donation pledging.

Variable	Category	OR	95% CI	*p*-value
Gender	Men (vs. Women)	0.506	0.413–0.620	<0.001
Generation	Gen X (vs. Baby boomers)	0.497	0.146–1.688	0.263
	Gen Y	0.641	0.391–1.053	0.079
	Gen Z	1.092	0.775–1.539	0.613
Education	Primary school (vs. PhD)	1.288	0.533–3.116	0.574
	Secondary school	0.503	0.203–1.248	0.138
	High school	0.557	0.240–1.293	0.173
	University	0.426	0.186–0.976	0.044
	Master	0.599	0.233–1.541	0.288
Marital status	Married (vs. Single)	0.667	0.453–0.984	0.041
Have children	Yes (vs. No)	1.161	0.762–1.769	0.486
Chronic disease	Yes (vs. No)	0.662	0.501–0.874	0.004
Health professional	Yes (vs. No)	1.863	1.414–2.456	<0.001
Health professional relative	Yes (vs. No)	1.046	0.847–1.292	0.674

OR, odds ratio; CI, confidence interval. Reference categories were women (gender), baby boomers (generation), PhD (education), single (marital status), no children, no chronic disease, non-health professional, and no health professional relative. Statistically significant associations (*p <* 0.05) indicate independent predictors of organ donation pledging.

### Univariate analysis of factors associated with organ donation pledging

Of the total participants (*n =* 16,738), 625 individuals (3.7%) reported having pledged to donate their organs, whereas 16,113 (96.3%) had not. Gender differences were statistically significant (*χ*^2^ = 15.232, *p <* 0.001, ES = 0.030), with a higher proportion of organ donation pledges observed among men (4.5%) than women (3.3%). Generational differences were also significant (*χ*^2^ = 361.347, *p <* 0.001, ES = 0.146). The highest pledge rate was observed in Generation X (10.8%), followed by baby boomers (7.4%) and Generation Y (6.2%), whereas the lowest rate was found in Generation Z (2.1%). Educational level was significantly associated with organ donation pledging (*χ*^2^ = 268.114, *p <* 0.001). The proportion of individuals who pledged increased with higher education levels, reaching the highest rates among PhD (16.3%) and master’s degree holders (13.4%) (Table 5).

**Table 5:** Sample characteristics of those who have donated organs.

Characteristics	*N* (%)	Have you pledged to donate your organs?		*χ* ^2^	*P* (ES)
		Yes	No		
Gender					
Men	6,181 (36.9%)	277 (4.5%)	5,904 (95.5%)	15.232	<0.001 (0.030)
Women	10,557 (63.1%)	348 (3.3%)	10,209 (96.7%)		
Generations					
Baby boomers (1946–1964)	121 (0.7%)	9 (7.4%)	112 (92.6%)	361.347	<0.001 (0.146)
Generation X (1965–1980)	1,531 (9.1%)	165 (10.8%)	1,366 (89.2%)		
Generation Y (1981–1996)	3,371 (20.1%)	208 (6.2%)	3,163 (93.8%)		
Generation Z (1997–2009)	11,715 (70%)	243 (2.1%)	11,472 (97.2%)		
Last graduation					
Primary School	662 (4%)	25 (3.8%)	637 (96.2%)	268.114	<0.001 (0.126)
Secondary School	1,321 (7.9%)	33 (2.5%)	1,288 (97.5%)		
High School	9,197 (54.9%)	211 (2.3%)	8,986 (97.7%)		
University	4,996 (29.8%)	278 (5.6%)	4,718 (94.4%)		
Master	464 (2.8%)	62 (13.4%)	402 (86.6%)		
PhD	98 (0.6%)	16 (16.3%)	83 (83.7%)		
Marital status					
Married	4,396 (26.3%)	324 (7.4%)	4,072 (92.6%)	219.306	<0.001 (0.114)
Single	12,342 (73.7%)	301 (2.4%)	12,041 (97.6%)		
Have children					
Yes	3,854 (23%)	296 (7.7%)	3,558 (92.3%)	216.919	<0.001 (0.113)
No	12,884 (77%)	329 (2.6%)	12,555 (97.4%)		
Have a chronic disease					
Yes	1955 (11.7%)	146 (7.5%)	1809 (92.5%)	85.860	<0.001 (0.071)
No	14,783 (88.3%)	479 (3.2%)	14,304 (96.8%)		
Are you a health professional					
Yes	2079 (12.4%)	162 (7.8%)	1917 (92.2%)	108.760	<0.001 (0.080)
No	14,659 (87.6%)	463 (3.2%)	14,196 (96.8%)		
Have a health professional relative					
Yes	9,836 (58.8%)	396 (4.0%)	9,440 (96%)	5.658	0.017 (0.018)
No	6,902 (41.2%)	229 (3.3%)	6,673 (96.7%)		
Total	16,738 (100%)	625 (3.7%)	16,113 (96.3%)		

Marital status was significantly associated with pledging (*χ*^2^ = 219.306, *p <* 0.001, ES = 0.114), with married participants showing a higher pledge rate (7.4%) compared to single participants (2.4%). Similarly, participants with children had higher pledge rates (7.7%) than those without children (2.6%) (*χ*^2^ = 216.919, *p <* 0.001, ES = 0.113). Participants with chronic diseases reported higher pledge rates (7.5%) than those without chronic diseases (3.2%) (*χ*^2^ = 85.860, *p <* 0.001, ES = 0.071). Being a health professional was also significantly associated with higher pledge rates (7.8% vs. 3.2%) (*χ*^2^ = 108.760, *p <* 0.001, ES = 0.080). Finally, having a health professional relative showed a statistically significant association (*χ*^2^ = 5.658, *p* = 0.017, ES = 0.018), with slightly higher pledge rates among those with such relatives (4.0%) than among those without (3.3%).

### Multivariable analysis of factors associated with organ donation pledging

To identify independent predictors of organ donation pledging, a multivariable logistic regression analysis was performed (Table 6). The overall model was statistically significant (*χ*^2^ = 508.948, *p <* 0.001) and demonstrated acceptable goodness-of-fit (Hosmer–Lemeshow *p* = 0.131). After adjustment, generation remained a strong independent predictor, with higher odds observed for Generation X, Generation Y, and Generation Z compared to baby boomers. Gender was also significantly associated with pledging, with lower odds observed among men. Furthermore, having a chronic disease and being a health professional were associated with a reduced likelihood of pledging. Conversely, marital status, having children, and having a health professional relative were no longer significant after adjustment (*p* > 0.05).

**Table 6:** Multivariable logistic regression analysis of factors associated with organ donation pledging.

Variable	Category	OR	95% CI	*p*-value
Gender	Men (vs. Women)	0.756	0.639–0.895	0.001
Generation	Gen X (vs. Baby boomers)	2.619	1.230–5.576	0.013
	Gen Y	3.781	2.737–5.225	<0.001
	Gen Z	1.928	1.488–2.498	<0.001
Education	Primary school (vs. PhD)	0.317	0.157–0.638	0.001
	Secondary school	0.443	0.226–0.867	0.018
	High school	0.591	0.325–1.072	0.083
	University	0.967	0.543–1.723	0.909
	Master	1.480	0.795–2.755	0.216
Marital status	Married (vs. Single)	0.792	0.599–1.049	0.104
Have children	Yes (vs. No)	0.868	0.644–1.170	0.353
Chronic disease	Yes (vs. No)	0.559	0.456–0.685	<0.001
Health professional	Yes (vs. No)	0.440	0.358–0.541	<0.001
Health professional relative	Yes (vs. No)	1.027	0.863–1.222	0.767

OR, odds ratio; CI, confidence interval. Reference categories were women (gender), baby boomers (generation), PhD (education), single (marital status), no children, no chronic disease, non-health professional, and no health professional relative. Statistically significant associations (*p <* 0.05) indicate independent predictors of organ donation pledging.

### The importance of body and organ donation

**Figure 1:**
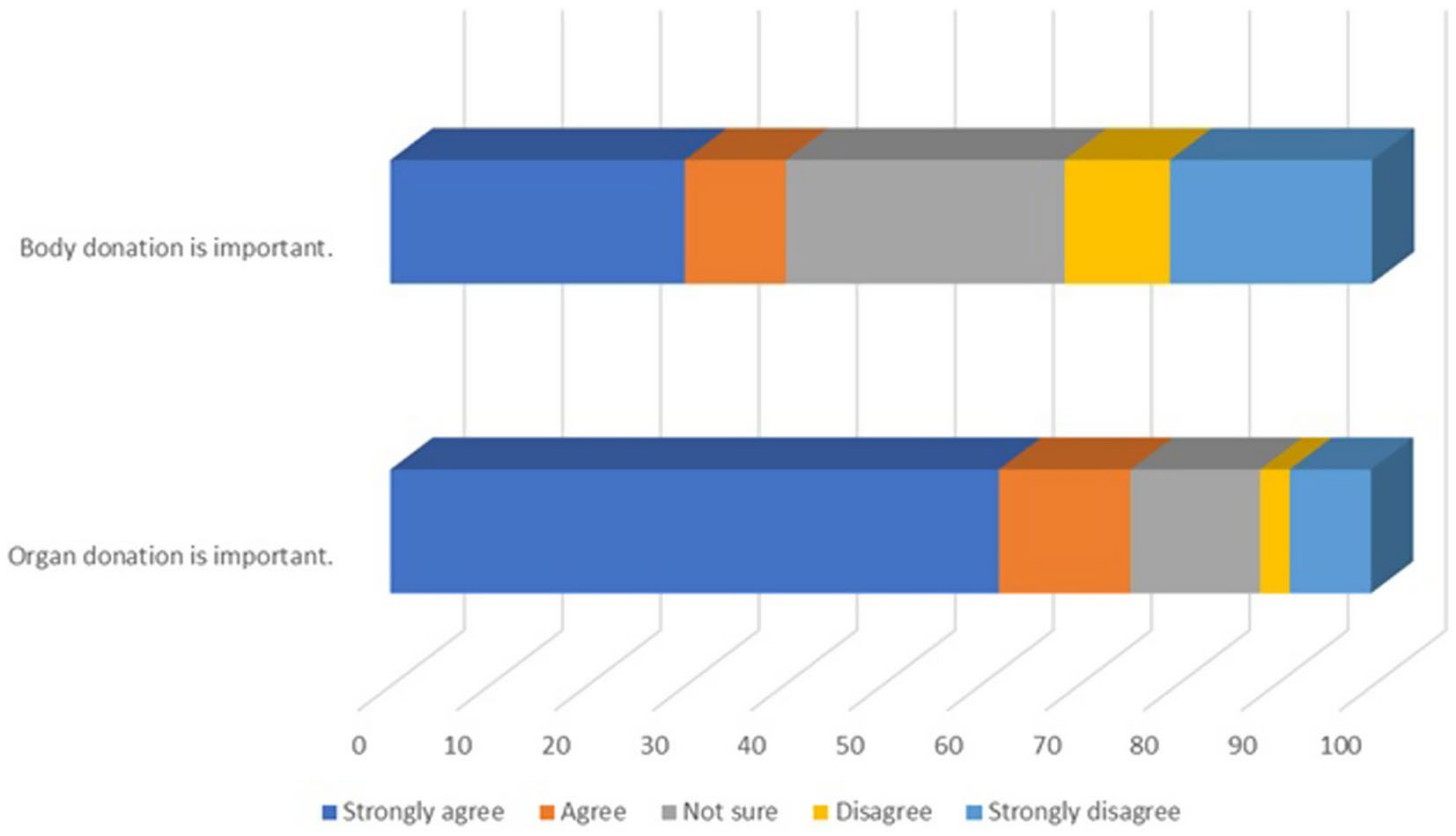
The importance of body and organ donation.

Participants’ attitudes toward organ and body donation were assessed using two statements: “Organ donation is important” and “Body donation is important.” Responses were recorded on a Likert scale from “strongly agree” to “strongly disagree.” The results are presented in Figure 1.

Organ donation was widely acknowledged as important, with most participants strongly agreeing. Conversely, the lower agreement for body donation underscores the need for targeted efforts to emphasize its critical role in medical education and research.

### Analysis of factors influencing willingness and unwillingness toward organ donation

Among participants willing to donate their organs (*n =* 9,845), the most commonly cited reason was “to save someone’s life” (74.7%), followed by “to serve humanity/help others” (56.5%). A significant number (38%) indicated personal experiences, such as having a relative in need of an organ transplant, as a motivating factor. Furthermore, psychological motivations such as leaving a legacy (“a piece of me will keep living after death,” 24.5%) were also notable (Table 7).

**Table 7:** Factors influencing willingness and unwillingness toward body and organ donation.

Reasons for body donation for those who want to donate their bodies* (*N*: 3052)	*N*	*P* (%)	Reasons for not donating organs or a body for those who do not want to donate a body* (*N:* 13686)	*N*	*P* (%)
Contribute to health education	2045	67	The thought of exposing their body	6,863	50.1
To feel more useful	1,469	48.1	It is not appropriate on religious grounds	4,416	32.2
The body becomes nonfunctional after death.	1,309	42.8	It is not appropriate according to their families	4,289	31.3
Do not want to be buried	206	6.7	Students disrespecting cadavers	3,638	26.5
Finding the thought of ceasing to exist scary	198	6.4	The thought that their body will suffer	2,571	18.7

Reasons for donating organs for those who want to donate organs* (*N*: 9845)	*N*	%	Reasons for not donating organs for those who do not want to donate organs* (*N*: 6893)	*N*	%
To save someone’s life	7,357	74.7	They do not want their body to lose its integrity	2,831	41.0
To serve humanity/Help others	5,568	56.5	Hesitant about organ donation due to a lack of information about it	1917	27.8
A relative who needs an organ donation	3,743	38.0	It is not appropriate on religious grounds	1882	27.3
The organs, which do not immediately rot after death. May be useful	3,270	33.2	The idea of donation reminds one of death	1,238	17.9
To serve science	2,510	25.4	My organs can be exploited commercially	1,143	16.5
A piece of me will keep living after death	2,413	24.5	They may leave me for dead/the fear of having organs-tissues removed before you completely die	985	14.2
Religious beliefs	961	9.7	The organs can be transplanted to someone whom the person does not want them transplanted to	662	9.6
To get rid of the feeling of guilt (compensating for the bad things I had done when I lived)	621	6.3	The thought that the organs could be used for medical research instead of donation	494	7.1

*Multiple selections possible.

For those unwilling to donate organs (*n =* 6,893), the primary barrier was concern about body integrity (41%), followed by insufficient information about the process (27.8%) and religious objections (27.3%).

### Analysis of factors influencing willingness and unwillingness toward body donation

Participants willing to donate their bodies (*n =* 3,052) emphasized contributions to health education (67.0%) as the primary motivator, reflecting an awareness of the educational importance of cadavers in medical training. Others mentioned the desire “to feel more useful” (48.1%) and the belief that the body becomes nonfunctional after death (42.8%) (Table 7).

Conversely, among those unwilling to donate their bodies (*n =* 13,686), the most frequently cited reason was discomfort with the idea of body exposure (50.1%). Religious objections (32.2%) and concerns about lack of family approval (31.3%) were also prominent.

### Sources and levels of awareness on organ and body donation

In our study on organ and body donation, we included a question to assess participants’ awareness and knowledge of these topics. Specifically, we asked whether they had any information about organ and body donation and the primary sources of this information. The responses were categorized and are shown in Table 8.

**Table 8:** Participants’ information on organ and body donation.

Participants’ information on body and organ donation		*N*	%
I know what/where to do to donate my body	Yes	4,914	29.4
	No	11,824	70.6
I received information from	No information	8,508	50.8
	Media (television, internet, social media)	4,182	25.0
	Health care personnel	1,563	9.3
	Hospital	1,446	8.6
	Anatomy Department	872	5.2
	Other	167	1.0.0
I know what/where to do to donate an organ	Yes	9,575	57.2
	No	7,163	42.8
I received information from	Media (television, internet, social media)	6,490	38.8
	No information	3,973	23.7
	Health care personnel	2,235	13.4
	Hospital	2,221	13.3
	Organ donation centers	1,497	8.9
	Other	322	1.9

The findings revealed a notable disparity in awareness levels. While 57.2% of participants knew where and how to donate organs, only 29.4% were aware of the procedures for body donation. Media (38.8%) was the most frequently cited source of information for organ donation, followed by healthcare personnel (13.4%) and hospitals (13.3%). Conversely, for body donation, 50.8% of participants reported having no information at all, with anatomy departments cited as a source by only 5.2%.

### Recommendations for promoting organ and body donations

To enhance organ and body donation rates, it is critical to understand public perspectives on effective strategies. Participants in our study provided valuable recommendations to increase awareness and encourage participation. These responses, categorized and summarized in Table 9, highlight key areas for intervention.

**Table 9:** Recommendations for promoting body and organ donations.

Recommendations		*N*	%
Recommendations to Promote Body Donation*	Educating society through visual and written media	7,553	45.1
	Incorporating the subject throughout all stages of schooling	6,256	37.4
	Promotional campaigns for body donation	5,036	30.1
	Clerics should inform society	4,675	27.9
	Healthcare personnel should conduct studies to promote organ donation	4,250	25.4
	Providing financial support to those who donate their bodies	3,618	21.6
	Making new legislative regulations governing the transplantation of cadavers from unclaimed bodies	3,384	20.2
Recommendations to Promote Organ Donation*	Incorporating the subject throughout all stages of schooling	7,710	46.1
	Educating society through visual and written media	7,651	45.7
	Promotional campaigns for organ donation	6,472	38.7
	Clerics should inform society	5,566	33.3
	Healthcare personnel should conduct studies to promote organ donation	4,682	28.0
	Making new legislative regulations governing the transplantation of organs from patients who have been declared brain dead	4,036	24.1
	Providing financial support to those who donate their organs	3,957	23.6

*Multiple selections are possible.

Educational initiatives were the top recommendation, with 46.1% of participants emphasizing the need to incorporate donation topics throughout all stages of schooling. Similarly, educating society through visual and written media was a widely suggested strategy (45.7% for organ donation, 45.1% for body donation). Promotional campaigns and the involvement of clerics in raising awareness were also frequently mentioned. For organ donation, legislative reforms (24.1%) and financial incentives (23.6%) were identified as additional strategies to address barriers.

### Attitudes toward relatives donating bodies

**Figure 2:**
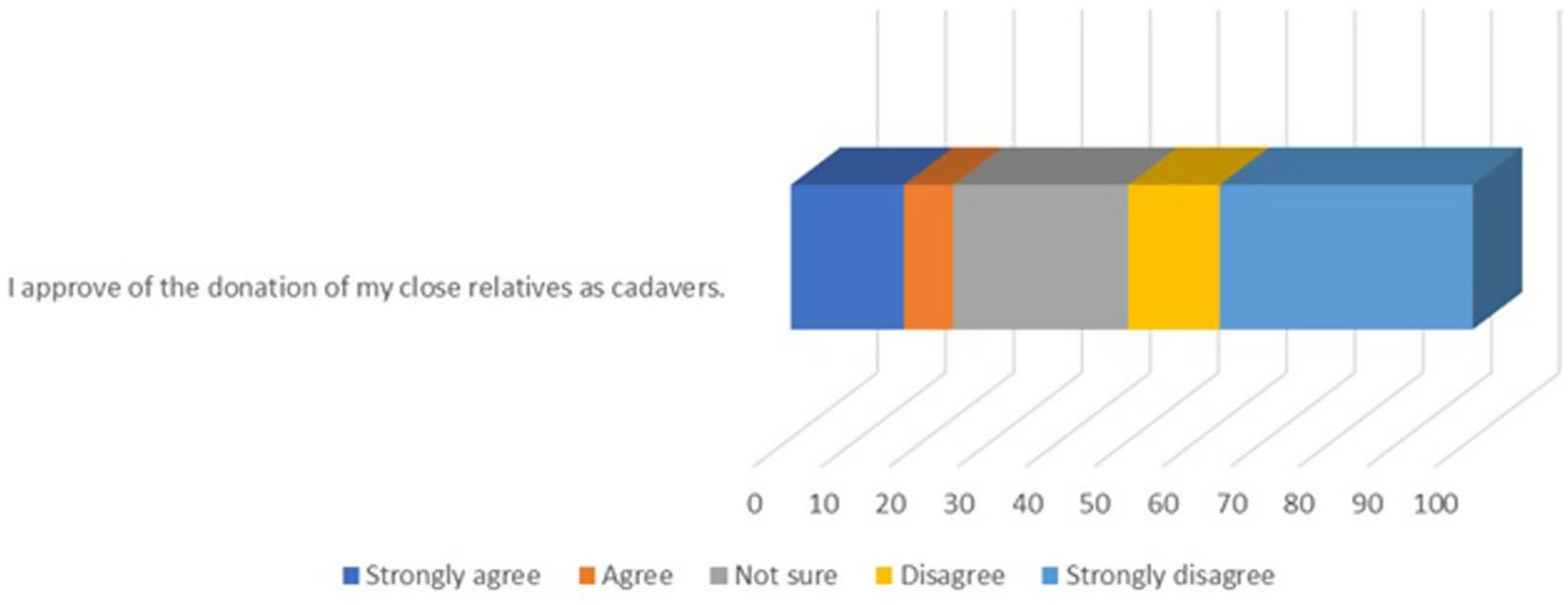
The rate of agreement with the statement “I approve of the donation of my close relatives as cadavers.

To examine societal acceptance of body donation, we asked participants about their attitudes toward a close relative donating their body. The question was phrased as: “Would you approve of your relative’s body donation?” Participants responded using a Likert scale ranging from “strongly agree” to “strongly disagree.” The responses are depicted in Figure 2.

The results reveal a significant divide in public opinion. While a portion of participants agreed with the idea, a notable percentage either disagreed or strongly disagreed, indicating cultural or emotional barriers within families. Neutral responses (“Not sure”) also accounted for a significant share, reflecting uncertainty or lack of awareness about the implications of body donation.

### Attitudes toward health professionals’ anatomy training

**Figure 3:**
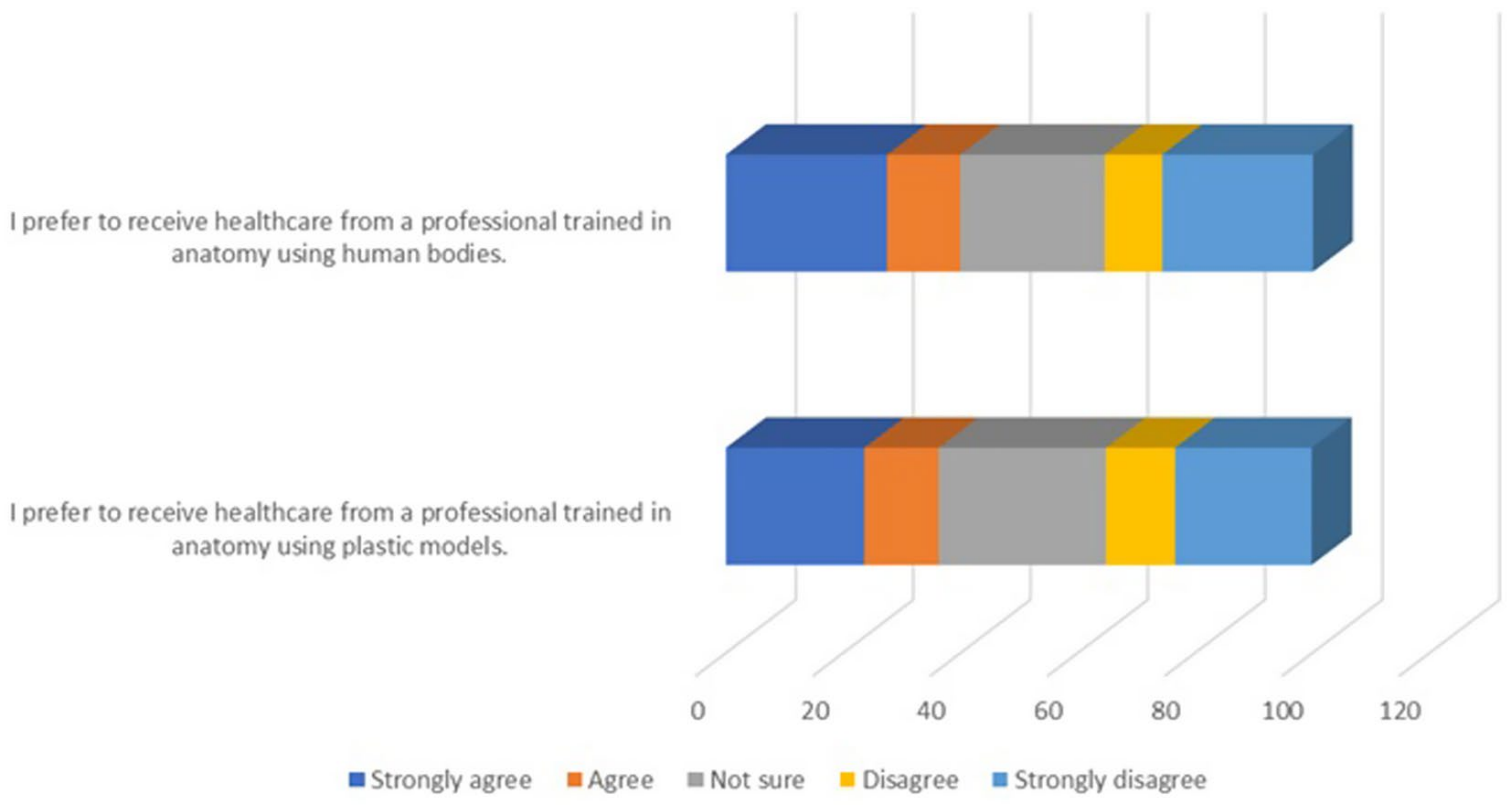
Preference for healthcare professionals based on anatomy training material.

In our survey, we asked a unique question not found in other studies: “I prefer to receive healthcare from a professional trained in anatomy using cadavers,” and “I prefer to receive healthcare from a professional trained in anatomy using plastic models.” Participants were asked to indicate the extent to which they agreed with these statements. The responses are illustrated in Figure 3.

The results revealed a higher preference for healthcare professionals trained using cadavers compared to plastic models. However, a significant proportion of participants selected “Not sure,” reflecting limited awareness of the impact of training methods. Those who agreed or strongly agreed with cadaver-based training emphasized its value in providing a deeper understanding of human anatomy.

## Discussion

This study provides a comprehensive and large-scale evaluation of public perspectives on body and organ donation in Türkiye, offering insights into the complex interplay among motivations, barriers, and awareness-related factors. While previous studies in this field have often relied on similar survey-based approaches, the present study distinguishes itself through its exceptionally large sample size (*n =* 16,738) and inclusion of diverse sociodemographic groups, thereby enhancing the robustness and generalizability of the findings.

The study by Boduç and Allahverdi (6) highlights a significant disparity in how medical students perceive organ and body donation, with organ donation being valued more highly than body donation (8). A similar trend was evident in our findings: the general population expressed stronger support for organ donation, whereas attitudes toward body donation were notably weaker. Conversely, medical students demonstrated greater appreciation for both types of donation, likely reflecting their exposure to medical education and recognition of the importance of anatomical studies. These results highlight the differences in awareness and attitudes across professional and non-professional groups, suggesting that targeted educational interventions tailored to different demographics and professional backgrounds may enhance support for both body and organ donation.

In our cohort, only 2.3% of participants had pledged to donate their bodies, with significant demographic differences. Men participants were more likely to pledge than women (3.3% vs. 1.8%, *χ*^2^ = 36.034, *p <* 0.001). Pledge rates varied across generations (*χ*^2^ = 8.589, *p* = 0.036), with relatively higher rates observed in Generation Y (3.0%), Generation X (2.4%), and baby boomers (2.5%) compared to Generation Z (2.1%). Educational level showed a significant association with pledging (*χ*^2^ = 34.436, *p <* 0.001), with the highest rates observed among PhD holders (7.1%) and master’s graduates (3.2%). However, this pattern was not strictly linear across all education levels. Married individuals were significantly more likely to pledge than singles (3.0% vs. 2.1%, *χ*^2^ = 12.422, *p <* 0.001). Higher pledge rates were also observed among participants with children (2.9% vs. 2.2%, *χ*^2^ = 5.893, *p* = 0.015) and those with chronic diseases (3.4% vs. 2.2%, *χ*^2^ = 11.551, *p* = 0.001). Professional background was also associated with pledging, as health professionals reported higher rates than non-professionals (3.7% vs. 2.1%, *χ*^2^ = 19.464, *p* = 0.001). Conversely, having a health professional relative was not significantly associated with pledging (*χ*^2^ = 0.921, *p* = 0.337). Overall, these findings indicate that body donation pledging was more frequently observed among men, certain age groups, individuals with higher education levels, married participants, and those working in healthcare.

Similarly, only 3.7% of participants had pledged to donate their organs, with several demographic differences reaching statistical significance. Men participants showed a higher pledge rate than women (4.5% vs. 3.3%, *p <* 0.001). Pledging varied significantly across generations (*p <* 0.001), with the highest rates observed in Generation X (10.8%) and baby boomers (7.4%), and the lowest in Generation Z (2.1%). Education was strongly associated with pledging: while 3.8% of primary school graduates had pledged, the rate increased to 13.4% among those with a master’s degree and 16.3% among PhD holders (*p <* 0.001). Marital status also influenced pledging, with married individuals reporting a higher rate (7.4% vs. 2.4%, *p <* 0.001). Health-related characteristics showed similar patterns, as those with chronic disease (7.5%) and health professionals (7.8%) had higher pledge rates than their counterparts (*p <* 0.001 for both). Having a health professional relative was also associated with slightly higher pledging rates (4.0% vs. 3.3%, *p* = 0.017). Overall, these findings indicate that organ donation pledging was more frequent among older generations, individuals with higher education levels, married participants, and those with links to the healthcare field.

The multivariable analyses provided a more robust understanding of donation behavior by identifying independent predictors after controlling for potential confounders. Several variables that were significant in univariate analyses, including marital status, having children, and having a health professional relative, lost their significance in the adjusted models, indicating that their apparent effects were largely explained by other sociodemographic factors. This finding underscores the importance of multivariable approaches in avoiding misleading interpretations based on simple comparisons. Conversely, gender, generation, and education remained significant predictors, although their effects differed in magnitude and direction across models. These results suggest that donation behaviors are influenced by a complex interplay of demographic and social factors rather than single isolated variables.

One of the most striking findings of this study is the divergent role of healthcare-related factors in organ and body donation. While being a health professional was associated with significantly lower odds of pledging organ donation, it was linked to higher odds of pledging body donation. This contrast may reflect differences in perception and familiarity, as healthcare professionals are more directly exposed to the educational and scientific value of cadaveric donation. In comparison, organ donation decisions may be influenced by broader emotional, cultural, and systemic factors that extend beyond professional knowledge. Furthermore, the absence of an independent effect of having a health professional relative suggests that direct professional experience plays a more critical role than indirect exposure. These findings highlight that organ and body donation should not be treated as interchangeable constructs and that tailored strategies are needed to address their distinct determinants.

In our cohort, 18.0% of participants expressed willingness to donate their bodies for education or research (men 19.9%; women 17.4%). Willingness varied across generations, being highest among Generation Z (19.6%) and Baby Boomers (19.0%), and increased consistently with education (from 12.5% among primary school graduates to 28.6% among PhD holders). Singles and participants without children were more willing than their married or parenting counterparts (all *p <* 0.001). Neither chronic disease status (*p* = 0.126) nor being a health professional (*p* = 0.227) was significantly associated with willingness, whereas having a health-professional relative showed a modest positive association (*p* = 0.010). The substantially lower acceptance compared with organ donation is consistent with international evidence. In Türkiye, willingness for body donation has been reported at ~26% compared to about ~69% for organ donation (10). Among Australian anatomy students, support for self-body donation (26.5%) lagged behind organ donation (82.5%) (11), whereas a population-based study in China found 27.5% willingness, with traditional beliefs (e.g., Confucian body integrity) and limited knowledge cited as key deterrents (12). Even within the anatomy community, Turkish anatomists reported psychological discomfort, anticipated family opposition, and concerns about disrespect as reasons not to donate their own bodies (7). On the system side, insufficient guidance from health professionals can further hinder potential donors; a national survey revealed that many Turkish physicians lacked basic procedural knowledge about body donation and expressed the need for training (13). Taken together, these findings reflect a global gap between organ and body donation and underscore the importance of culturally sensitive outreach and clearer procedural pathways to reduce reluctance.

Conversely, 58.8% of participants expressed willingness to donate their organs after death (men 51.8%, women 63.0%). Willingness was higher among younger cohorts, rising from 44.6% among Baby Boomers to 60.8% among Generation Z, and increased steadily with education (from 37.0% among primary school graduates to 78.6% among PhD holders). Singles and participants without children were more willing than married individuals and parents (all *p <* 0.001). Both being a health professional and having a relative who is in a health profession were associated with higher willingness (*p <* 0.001 for both), whereas chronic disease status showed no significant effect (*p* = 0.158). This pattern—organ donation being widely accepted and increasing with education and knowledge—parallels prior findings. For example, Turkish university employees reported greater readiness for organ than body donation (69.1% vs. 26.2%) (10). Turkish medical students likewise showed high willingness (≈71%) (14), whereas Indian medical and nursing students endorsed organ donation despite knowledge gaps that targeted training could address (15). Broader Turkish surveys also emphasize that willingness is generally common but tempered by concerns about body integrity and religion, underscoring the importance of public education and family discussion (6).

Within our cohort, the most prominent reasons for unwillingness to donate were religious concerns, insufficient knowledge, and the belief that bodily integrity would be disturbed. Similar barriers have been reported elsewhere: both Turkish and Saudi samples identified “religious inappropriateness” and “insufficient knowledge” as major deterrents (6, 16). A nationwide study in China emphasized bodily integrity as the primary obstacle (17). Research in Israel highlighted fear of post-mortem disfigurement and distrust of the brain death concept (18). Overall, these findings illustrate that culturally and religiously rooted concerns represent persistent global barriers to donation.

Conversely, the leading motivations for willingness to donate organs in our sample were “saving lives” and “helping others.” Similar drivers have been documented in other settings. Studies from Türkiye also identified altruism and serving humanity as key reasons for donation (6). A validation study in Saudi Arabia confirmed that the desire to “save lives” was the most influential factor (16). Research from Iran linked higher altruism scores with more favorable attitudes (19). Furthermore, a study in Israel concluded that although altruism was important, positive attitudes more strongly predicted willingness (18). Overall, our findings are consistent with these international trends, reinforcing the central role of altruism and prosocial values in shaping willingness.

In our study, the most common reasons for body donation were contributing to medical education, feeling useful after death, and the belief that the body becomes nonfunctional after death. These motives closely align with international evidence. In France, medical tutors emphasized that dissection of donated bodies is essential for anatomy education and for shaping ethical and professional values (20). Studies from China similarly highlighted the role of body donation in advancing medical education and scientific progress, portraying donors as “silent mentors” who contribute to the training of future physicians (21). Italian research reported that a majority of medical students and faculty were willing to donate their bodies, primarily citing advances in science and education (22). Comparable findings were observed in Ghana and Uganda, where physicians and academics valued body donation as a contribution to medical science and a way of supporting future training (23, 24). Collectively, these parallels suggest that altruistic motives centered on education and scientific contribution are consistently recognized across different contexts, resonating strongly with our participants’ perspectives.

Conversely, the primary barriers to body donation in our sample included concerns about exposure of the body, perceived religious inappropriateness, lack of family approval, and fears of disrespectful treatment by students. Similar obstacles have been documented internationally. Chinese studies identified traditional values emphasizing bodily integrity and Confucian funeral practices as significant deterrents (21). Slovenian students reported reluctance due to concerns over cadaver treatment, particularly among those with strong religious affiliations (25). In Italy, religiosity was negatively associated with willingness, underscoring the enduring influence of spiritual beliefs despite supportive legal frameworks (22). Likewise, in Ghana and Uganda, cultural and religious reservations, alongside fears of body misuse, were leading reasons for refusal (23, 24) These consistent patterns demonstrate that participants’ hesitations reflect a broader global trend in which religious, cultural, and trust-based concerns frequently outweigh altruistic intentions in decisions about body donation.

Awareness of organ and body donation is influenced by cultural, religious, and educational factors, with key sources of information including donation centers, healthcare professionals, educational institutions, and media campaigns (26). Nevertheless, significant disparities persist. In our study, while 57.2% of participants knew where and how to donate organs, only 29.4% reported the same knowledge for body donation. Remarkably, only 5.2% of respondents had received information about body donation from anatomy departments, underscoring the limited effectiveness of these institutions in raising awareness. Similarly, only 8.9% reported receiving information about organ donation from organ donation centers.

These results are consistent with international evidence showing a general lack of awareness about both organ and body donation (12, 15, 27). Studies among Turkish medical students also highlight insufficient knowledge about body donation (28, 29). Interestingly, awareness of organ donation does not appear to differ significantly between medical students and the general population, although levels remain higher for organ donation than for body donation.

In this study, the media emerged as the primary source of information for both organ and body donation, surpassing healthcare professionals and hospitals. This finding is consistent with prior research identifying the media as the most influential channel for raising awareness (6, 12, 15, 26). However, the information disseminated through media outlets is often insufficient (28). To improve public education, media channels should be utilized more effectively. Television, social media, and public service announcements (PSAs) should form the core of awareness campaigns. While PSAs on organ donation are already broadcast in Türkiye, no comparable campaigns currently address body donation. Anatomy departments should therefore play a more active role by engaging the public via social media, and collaborations with the Ministry of Health could facilitate the production and distribution of body donation–specific PSAs.

Structural differences in registration processes may also contribute to the disparity between organ and body donation. In Türkiye, organ donation can be registered online through the e-Devlet system, making the procedure simple and accessible. Conversely, body donation requires an in-person visit to a medical school’s anatomy department to complete the necessary forms. Integrating body donation registration into the e-Devlet platform could remove procedural barriers, streamline communication between donors and anatomy departments, and ultimately strengthen public trust and participation.

Public suggestions in our study emphasized three key strategies to increase donations: raising awareness through media, integrating donation topics into all stages of education, and organizing promotional campaigns. Involving religious leaders was also frequently mentioned. These proposals align with the public’s recognition that education and campaigns are critical for overcoming barriers. Notably, the Presidency of Religious Affairs (Diyanet) has already issued fatwas confirming the permissibility of both organ and body donation under Islamic law (30, 31). Accordingly, future efforts should focus less on debating religious permissibility and more on informing the public about these rulings, thereby reinforcing education and awareness.

Education-related strategies received strong support. Research shows significant knowledge gaps among university students: for instance, 51.4% of medical and law students in one study had never attended a course, presentation, or seminar on the topic (29). Incorporating donation-related content into curricula and organizing seminars would help address this gap and foster awareness in younger generations. Promotional campaigns were also highlighted as effective, with the Anatomy Week campaign organized by TAKAD leading to an increase in body donations (3). Expanding such initiatives could further enhance awareness and connect with potential donors.

In Türkiye, the process of body donation involves several formal steps to ensure donor consent is properly documented. Donors must sign a form in the presence of two witnesses, with a faculty member from the Anatomy Department serving as a “third witness” before submission (Turkish Society of Anatomy and Clinical Anatomy). Following the donor’s death, relatives are responsible for notifying the university to initiate transfer procedures. However, if relatives fail to do so or oppose the donation, the body is buried instead. This underscores the critical role of relatives in either facilitating or preventing the realization of donor wishes. In this study, half of the participants disagreed or strongly disagreed with the statement, “I would approve if my relative donated their body,” highlighting a substantial barrier. Familial reluctance, influenced by cultural, emotional, and informational factors, poses a challenge to honoring donors’ intentions. Public campaigns must therefore emphasize family involvement, provide clear information on ethical safeguards, and encourage open discussions within families about donation decisions.

We also explored public perceptions of anatomy training by asking whether respondents would prefer healthcare professionals trained on cadavers versus plastic models. For cadaver-based training, 27.5% strongly agreed, and 12.5% agreed, whereas 24.6% were neutral, 9.8% disagreed, and 25.7% strongly disagreed. For plastic models, 23.6% strongly agreed, 12.7% agreed, 28.5% were neutral, 11.8% disagreed, and 23.3% strongly disagreed. These results suggest limited public awareness of the advantages of cadaver-based training. The high proportion of neutral responses reflects uncertainty and underscores the need for public education campaigns on the critical role of cadavers in developing clinical and anatomical competence. Such initiatives could increase support for body donation programs and help secure a sustainable supply of cadavers for education.

Overall, organ donation appears to be more widely accepted than body donation in Türkiye, although actual pledging remains limited for both. Donation behaviors are influenced by a complex interplay of educational, cultural, religious, and familial factors. Addressing these barriers requires increased awareness, transparent processes, and culturally sensitive communication strategies to build public trust.

## Limitations

This study has several limitations that should be considered when interpreting the findings. First, the use of snowball sampling may limit the generalizability of the results, as participants were recruited through existing social networks, potentially introducing selection bias and underrepresenting certain demographic groups in Türkiye. Furthermore, data were collected via self-reported measures, which may be influenced by social desirability bias. Although inferential analyses and multivariable logistic regression models were conducted to identify independent predictors of donation behaviors, the cross-sectional design precludes causal inferences. Furthermore, the use of an online survey platform (Google Forms) may have excluded individuals with limited internet access, particularly those from rural areas or older populations. Another limitation is that religious influences were not directly assessed using validated measures; instead, they were inferred from participants’ responses. Future research should incorporate standardized instruments to better capture the role of religion in shaping donation attitudes. Despite these limitations, the large sample size, methodological rigor, and inclusion of diverse sociodemographic variables strengthen the overall contribution of the study.

## Conclusion

This study demonstrated that although organ donation is generally accepted in Türkiye, body donation remains limited, with substantial gaps between willingness and actual pledging. Donation behaviors are influenced by a complex interplay of educational, cultural, religious, and family factors, with awareness and trust being key determinants.

The findings emphasize the importance of targeted, culturally sensitive strategies to improve both organ and body donation rates, including strengthening public education, increasing the visibility of donation processes, and enhancing the role of media, healthcare professionals, and religious authorities in addressing misconceptions.

Although the results should be interpreted with consideration of the study’s methodological limitations, the large sample size and comprehensive analytical approach provide valuable insights to guide future research and policy development aimed at increasing donation rates in Türkiye.

## Data Availability

The raw data supporting the conclusions of this article will be made available by the authors, without undue reservation.
