# Supplementary material for: Public perspectives on body and organ donation in Türkiye: barriers, motivations, and strategies for awareness
Source: Front Public Health. 2026 Apr 13;14:1801282. doi: 10.3389/fpubh.2026.1801282 (PMC13111388; doi:10.3389/fpubh.2026.1801282)
Supplement: Supplementary file 1 [file Supplementary_File_1.docx]

**Body and Organ Donation Questionnaire**

This survey has been designed to evaluate Türkiye’s Current Perspectives on Whole-Body and Organ Donation. The information and opinions you provide will contribute significantly to understanding the underlying reasons for the current insufficiency of body and organ donation. The questionnaire consists of 8 demographic questions and 17 items related to body and organ donation. Completing the survey will take approximately 5 minutes. Participation is entirely voluntary. Your responses will remain confidential, will be used exclusively for research purposes, and no personal information will be disclosed. Please do not include your name, surname, or address. You are free to decline participation or discontinue the survey at any time without any consequences. We sincerely thank you for your valuable contribution and for sharing your honest views, which will help us better understand challenges and potential solutions regarding body and organ donation.

**Descriptive Questions**

1. **Your birth year:**
2. **What is the highest level of education you have completed?**

Primary school

Middle school

High school

University

Master’s degree

Doctorate

1. **What is your gender?**

Female

Male

1. **Are you married?**

Yes

No

1. **Do you have children?**

Yes

No

1. **Do you have any chronic illnesses?**

Yes

No

1. **Are you a healthcare professional?**

Yes

No

1. **Do you have a relative who is a healthcare professional?**

Yes

No

**Organ Donation**

1. **"Organ donation is important." (Please rate your level of agreement)**

Strongly disagree (1)

Disagree (2)

Not sure (3)

Agree (4)

Strongly agree (5)

1. **Have you ever donated an organ?**

Yes

No

1. **Would you donate your organs?**

Yes (Proceed to "Why would you donate your organs?")

No (Proceed to "Why wouldn’t you donate your organs?")

1. **Why would you donate your organs? (You can select multiple options)**

I want to save someone’s life.

I want to serve humanity/help people.

I believe organs are nonfunctional after death.

I believe a part of me will live on after death.

I want to serve science.

I want to donate for religious reasons.

I would donate if a loved one needed an organ.

I want to make amends for the bad things I’ve done in life.

1. **Why wouldn’t you donate your organs? (You can select multiple options)**

I don’t want to lose my bodily integrity.

I don’t believe it is religiously appropriate.

I’m concerned my organs could be used for commercial purposes.

I fear they might let me die to harvest my organs.

Organ donation reminds me of death.

I think my organs could be used for medical research instead of donation.

I hesitate due to insufficient knowledge about organ donation.

I’m worried my organs might be donated to someone I wouldn’t want to receive them.

**Whole-Body Donation**

1. **"Whole-body donation is important." (Please rate your level of agreement)**

Strongly disagree (1)

Disagree (2)

Not sure (3)

Agree (4)

Strongly agree (5)

1. **Have you ever donated your body?**

Yes

No

1. **Would you donate your body?**

Yes (Proceed to "Why would you donate your body?")

No (Proceed to "Why wouldn’t you donate your body?")

1. **Why would you donate your body? (You can select multiple options)**

I want to contribute to health education.

I want to feel more useful.

I don’t want to be buried.

The thought of decaying terrifies me.

I believe my body will be nonfunctional after death.

1. **Why wouldn’t you donate your body? (You can select multiple options)**

My family wouldn’t approve.

I don’t think it is religiously appropriate.

I fear people may disrespect the cadavers.

I don’t want my body to be displayed.

I believe my body would feel pain.

**Sources and Levels of Awareness on Organ and Whole-Body Donation**

1. **Do you know how/where organ donation is performed?**

Yes

No

1. **Where did you learn about organ donation?**

Media (TV, internet, social media)

Healthcare professionals

Hospital

Organ donation center

I have no information

1. **Do you know how/where body donation is performed?**

Yes

No

1. **Where did you learn about body donation?**

Media (TV, internet, social media)

Healthcare professionals

Hospital

Department of Anatomy

I have no information

1. **"I would approve of a close relative donating their body" (Please rate your level of agreement)**

Strongly disagree (1)

Disagree (2)

Not sure (3)

Agree (4)

Strongly agree (5)

**Attitudes Toward Health Professionals' Anatomy Training**

1. **"I prefer receiving healthcare services from professionals who have been trained on human bodies in anatomy education."**

Strongly disagree (1)

Disagree (2)

Not sure (3)

Agree (4)

Strongly agree (5)

1. **"I prefer receiving healthcare services from professionals trained on plastic models instead of human bodies."**

Strongly disagree (1)

Disagree (2)

Not sure (3)

Agree (4)

Strongly agree (5)

**Recommendations for Promoting Organ and Whole-Body Donations**

1. **What is your solution to the lack of organ donation in Türkiye? (You can select multiple options)**

Informing the public through written and visual media

Including the topic in all levels of education

Campaigns organized by healthcare professionals to promote donation

Religious leaders informing the public

Organizing promotional campaigns for organ donation

New legal regulations on organ transplantation from brain-dead patients

Providing financial support to those who donate their organs

1. **What is your solution to the lack of body donation in Turkey? (You can select multiple options)**

Informing the public through written and visual media

Including the topic in all levels of education

Campaigns organized by healthcare professionals to promote donation

Religious leaders informing the public

Organizing promotional campaigns for body donation

New legal regulations regarding body donation from unclaimed individuals

Providing financial support to those who donate their bodies
